# Comparative karyotypic study of fifteen cyprinids (Cyprinidae, Cyprininae) species. An insight into the chromosomal evolution of the tribe Systomini

**DOI:** 10.1371/journal.pone.0305828

**Published:** 2024-07-18

**Authors:** Phichaya Buasriyot, Francisco de Menezes Cavalcante Sassi, Nuttasuda Donbundit, Weerayuth Supiwong, Sarawut Kaewsri, Sarun Jumrusthanasan, Kriengkrai Seetapan, Krit Pinthong, Alongklod Tanomtong, Klodthida Yanukun, Nawarat Muanglen, Thomas Liehr, Marcelo de Bello Cioffi

**Affiliations:** 1 Animals Cytogenetics Research Group, Department of Biology, Faculty of Science, Khon Kaen University, Muang, Khon Kaen, Thailand; 2 Departamento de Genética e Evolução, Universidade Federal de São Carlos (UFSCar), São Carlos, SP, Brazil; 3 Faculty of Interdisciplinary Studies, Nong Khai Campus, Khon Kaen University, Muang, Nong Khai, Thailand; 4 Biology Program, Department of Science, Faculty of Science, Buriram Rajabhat University, Muang, Buriram, Thailand; 5 School of Agriculture and Natural Resources, University of Phayao, Muang District, Phayao Province, Thailand; 6 Department of Fundamental Science, Faculty of Science and Technology, Surindra Rajabhat University, Muang, Surin, Thailand; 7 Department 0f Fisheries, Faculty of Agricultural Technology, Sakon Nakhon Rajabhat University, Muang, Sakon Nakhon, Thailand; 8 Institute of Human Genetics, University Hospital Jena, Friedrich Schiller University, Jena, Germany; Sher-e-Kashmir University of Agricultural Sciences and Technology of Kashmir, INDIA

## Abstract

The family Cyprinidae is the largest freshwater fish group with 377 genera and over 3,000 described species. However, this group of fish has very limited cytogenetics and advanced molecular cytogenetics information. Therefore, in this study the karyotypes and other chromosomal characteristics of 15 species in the tribe Systomini (Cyprininae) were examined using Ag-NOR staining along with fluorescence in situ hybridization (5S and 18S rDNA). All species share a similar karyotype (2n = 50; NF = 88–100) in both sexes and no differentiated sex chromosome was observed. Chromosomes bearing NOR sites ranged from one to four pairs among the species, mostly mapped adjacent to telomeres in the short arms of distinct pairs in all analyzed species. This difference indicates an extensive rearrangement of chromosomes including genomic differences. The use of the 5S and 18S rDNA probe confirmed the Ag-NOR sites interstitially located in the telomeric regions of distinct chromosomes, characterizing an interspecies variation of these sites. In most of its analyzed species, the signals of 18S rDNA probe corresponded to the Ag-NOR regions, except in *Barbonymus altus*, *B*. *gonionotus*, *B*. *schwanenfeldii* and *Puntius brevis* having these signals on the same as Ag-NOR regions and other sites.

## Introduction

Cyprinidae is the largest and most diverse fish family with about 3,000 species and 370 genera naturally distributed throughout most parts of the world [1]. More than 1,500 cyprinid species have evolved highly adapted body shapes and mouth structures, allowing them to live in almost any habitats throughout their range [2]. This group’s basic traits include that it only has teeth in its throat, a single dorsal fin, pelvic fins in an abdominal position, pectoral fins low on the side, and absence of adipose fin [3]. The scales are cycloid and usually absent in the head, whereas the lateral-line system is typically well developed. The size and shape of the arch and teeth are closely tied to the diet of the species. In most cyprinids the lips are usually narrow, but they can be enlarged, sucker-like, or even lobed. Most cyprinids have a typical minnow-like body shape and are sexually dimorphic [4]. In Thailand’s aquatic ecosystems, 36 to 39% of all fish species are cyprinids [5].

Cytogenetics is an important tool for the detection of biodiversity [6, 7]. Many cytogenetic studies have been performed to understand the evolution of the macro and micro karyotype structures of different fish groups. Together, classical and molecular cytogenetic techniques have contributed significantly to knowledge of this karyotypic diversity [8]. Classical chromosomal banding techniques include in fish the C-banding and silver nitrate staining to highlight nucleolar organizer regions (Ag-NORs) [8]. These methods can detect chromosomal rearrangements, structural and/or numeric polymorphisms, sex chromosome systems, and populational variations [7, 9]. Although these techniques provide a good understanding of the fish chromosomal diversity, conventional karyotyping is usually limited to detecting the DNA rearrangements greater than 5 Mb. The fluorescence in *situ* hybridization (FISH) technique strongly improved the transition from classical to molecular cytogenetics by allowing the identification of DNA sequences ranging in size from 100kb to 1Mb in the studied cytological material [10]. As a result, this technique enables for the mapping of specific nucleotide sequences in within chromosomes [11]. In the same way, the position of NORs in fish has been widely documented, and polymorphisms within and among them have been discovered [12–14]. These experiments used silver nitrate staining (Ag-NOR), which has recently been verified or reinvestigated in light of FISH with 18S rDNA probe [15]. Furthermore, some fish species have the 18S rRNA gene co-located with the 5S rRNA [16, 17], another ribosomal sequence frequently investigated regarding its position on chromosomes.

The study of chromosomes in Cyprinidae reveals that the karyotypic evolution in this family is marked by several polyploidization events occurred independently in many species [18]. A diploid number ranging from 2n *=* 42 in *Acheilognathus gracilis* [19] to 2n *=* 446 in *Diptychus dipogon* [20] is described for cypriniforms, but 2n = 50 is the most frequent number and is considered a plesiomorphic trait for the group [21–23]. Differentiated sex chromosomes seem rare, with only one ZZ/ZW case described for *Squalius recurvirostris* [24–26]. Recently, a study combining molecular and classical cytogenetics revealed that the sister tribe Labeonini have a conserved 2n but with extensive structural chromosome rearrangements [27]. Thus, the aim of the present study was to provide the first finer-scale cytogenetic investigation in cyprinids from the Systomini tribe using both by conventional (Giemsa staining and Ag-NOR) and molecular (fluorescence in situ hybridization (FISH) with 5S and 18S rDNA probes) methods in 15 species. The results added new informative characters useful in comparative genomics at the chromosomal level and highlighted the inner diversity present among the analyzed species.

## Materials and methods

### Sample collection and mitotic chromosome preparation

**Fig 1:**
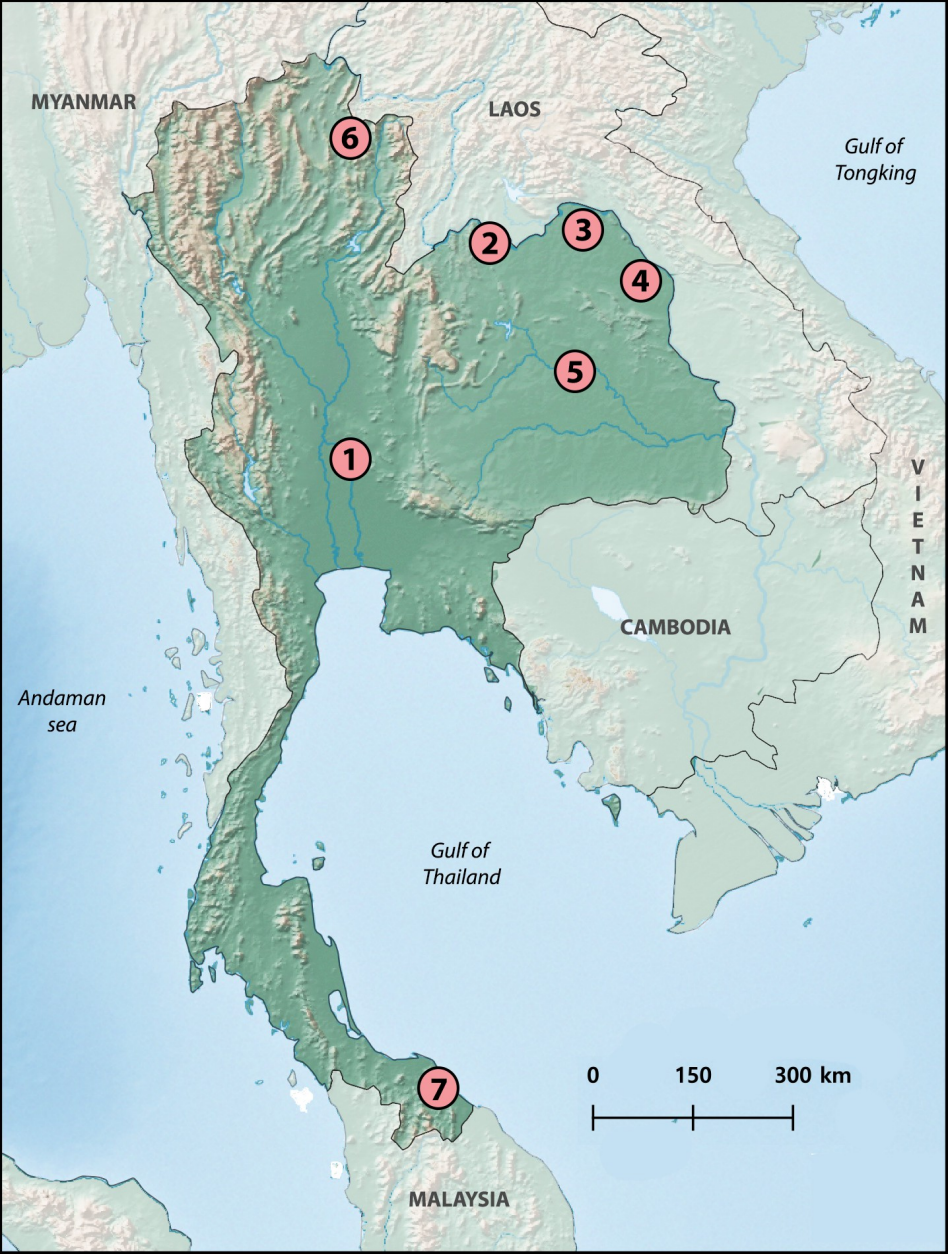
Thailand map indicating the seven collection sites of the 15 species studied herein. 1. *Amblyrhynchichthys micracanthus*, *Cyclocheilichthys armatus*, *Cyclocheilichthys repasson*, *Sikukia stejnegeri*; 2. *Cyclocheilos enoplos*, *Poropuntius laoensis*, *Puntius brevis*; 3. *Hampala macrolepidota*, *Barbonymus altus*, *Barbonymus schwanenfeldii*; 4. *Barbonymus gonionotus*, *Hampala dispar*; 5. *Puntigrus partipentazona*; 6. *Pethia stoliczkana* and 7. *Desmopuntius hexazona*. Map created with Natural Earth on QGIS 3.20. Scale Bar = 300 km.

Locations of sampling from the river basins in Thailand included the Chao Phraya River Basin (Sing Buri Province (1)) in the central region, the Mekong River Basin (Nong Khai Province (2)), Songkhram River Basin (Bueng Kan (3) and Nakhon Phanom Provinces (4)) Chi River Basin (Maha Sarakham Province (5)) in the northeastern region, Yom River Basin (Phayao Province (6)) in the northern region and Sirindhorn Peat Swamp Forest (To Daeng Peat Swamp Forest) (Narathiwat Province (7)) in the southern region of Thailand (Fig 1). Fifteen cyprinid species including *Amblyrhynchichthys micracanthus*, *Barbonymus altus*, *Barbonymus gonionotus*, *Barbonymus schwanenfeldii*, *Cyclocheilos enoplos*, *Cyclocheilichthys armatus*, *Cyclocheilichthys repasson*, *Desmopuntius hexazona*, *Hampala dispar*, *Hampala macrolepidota*, *Pethia stoliczkana*, *Poropuntius laoensis*, *Puntigrus partipentazona*, *Puntius brevis* and *Sikukia stejnegeri* were analyzed, with the number of individuals and sex compiled in Table 1. Mitotic chromosomes were obtained from the classical air-drying method, with some adaptations as described in previous works [28, 29]. The chromosome was stained with Giemsa’s solution pH 6.8 and the Ag-NOR banding was performed following the protocols of Howell and Black (1980) [30]; modified by [29, 31]. All experiments followed the scientific laboratory animal ethical conduct. This process has been approved by the Institutional Animal Care and Use Committee of Khon Kaen University, based on the Ethics of Animal Experimentation of the National Research Council of Thailand, record no. IACUC-KKU-40/64 and by the Royal Golden Jubilee (RGJ) committee under no. PHD/0169/2560 (Thailand).

**Table 1:** Collection sites and the number of specimens for chromosomal analysis.

*Species*	*Location*	*No. of specimens*
1. *Amblyrhynchichthys micracanthus*	Chao Phraya River Basin	07♀ 09♂
	14°51’30”N 100°24’42”E Ton Pho, Mueang Sing Buri District, Sing Buri	
2. *Barbonymus altus*	Songkhram River Basin	11♀; 08♂
	18°00’20.4"N 103°28’23.6"E	
	So Phisai District, Bueng Kan	
3. *Barbonymus gonionotus*	Songkhram River Basin	06♀;06♂
	17°43’12.0"N 104°06’55.9"E Sam Phong, Si Songkhram District, Nakhon Phanom	
4. *Barbonymus schwanenfeldii*	Songkhram River Basin	10♀:07♂
	18°00’20.4"N 103°28’23.6"E	
	So Phisai District, Bueng Kan	
5. *Cyclocheilos enoplos*	Mekong River Basin	12♀;15♂
	17°52’42.0"N 102°43’07.1"E Mi Chai, Mueang Nong Khai District, Nong Khai	
6. *Cyclocheilichthys armatus*	Chao Phraya River Basin	08♀;11♂
	14°51’30”N 100°24’42”E	
	Ton Pho, Mueang Sing Buri District, Sing Buri	
7. *Cyclocheilichthys repasson*	Chao Phraya River Basin	04♀;10♂
	14°51’30”N 100°24’42”E Ton Pho, Mueang Sing Buri District, Sing Buri	
8. *Desmopuntius hexazona*	To Daeng Peat Swamp Forest	05♀;06♂
	6°04’31”N 101°57’45”E	
	Puyo, Su-ngai Kolok District, Narathiwat	
9. *Hampala dispar*	Songkhram River Basin	09♀;10♂
	17°43’12.0"N 104°06’55.9"E Sam Phong, Si Songkhram District, Nakhon Phanom	
10. *Hampala macrolepidota*	Songkhram River Basin	09♀;09♂
	18°00’20.4"N 103°28’23.6"E	
	So Phisai District, Bueng Kan	
11. *Pethia stoliczkana*	Yom River Basin	08♀;12♂
	18°54’07.0"N 100°16’30.0"E Chiang Muan, Chiang Muan District, Phayao	
12. *Poropuntius laoensis*	Mekong River Basin	10♀;07♂
	17°52’42.0"N 102°43’07.1"E	
	Mi Chai, Mueang Nong Khai District, Nong Khai	
13. *Puntigrus partipentazona*	Chi River Basin	11♀;09♂
	16°13’55.2"N 103°15’59.0"E Tha Khon Yang, Kantharawichai District, Maha Sarakham	
14. *Puntius brevis*	Mekong River Basin	07♀;05♂
	17°52’42.0"N 102°43’07.1"E	
	Mi Chai, Mueang Nong Khai District, Nong Khai	
15. *Sikukia stejnegeri*	Chao Phraya River Basin	08♀;09♂
	14°51’30”N 100°24’42”E Ton Pho, Mueang Sing Buri District, Sing Buri	

### FISH analysis

Two probes were mapped using fluorescence in situ hybridization (FISH) in the mitotic metaphases. The 5S rDNA probe consisted of 120 base pairs (bp) of the 5S rRNA-encoding gene and 200 bp of the non-transcribed spacer (NTS) [32]. On other hand, the 18S rDNA probe corresponded to a 1400 bp segment of the 18S rRNA gene [33]. Both 5S and 18S rDNA probes were fluorescence labeled with the Nick-Translation Labeling Kit (Jena Bioscience, Jena, Germany) by Atto488-dUTP (18S rDNA) and Atto550-dUTP (5S rDNA), according to the manufacturer’s recommendations.

FISH was performed under high stringency conditions following the protocol of [34]. The chromosome preparations were incubated with RNAse (40 μg/mL) for 1.5 h at 37 ˚C. After denaturation of the chromosomes for 3 min in 70% formamide/2× SSC at 70 ˚C, spreads were dehydrated in an ethanol series (70, 85, and 100%), for 2 min each. Then, 20 μL of the hybridization mixture (100 ng of each probe, 50% deionized formamide, 10% dextran sulfate) was applied to the slides, and the hybridization was performed overnight at 37 ˚C in a moist chamber containing 2× SSC. The post-hybridization wash was carried out with 1× SSC for 5 min at 65 ˚C and a final wash was performed at room temperature in 4× SSC/Tween for 5 min. Finally, the chromosomes were counterstained with DAPI mounted in an antifade solution (Vectashield from Vector laboratories).

### Cytogenetic analysis

Approximately 30 metaphase spreads were analyzed per individual to confirm the diploid chromosome number (2n), karyotype structure, and FISH results. Images were captured using an Axioplan II microscope (Carl Zeiss Jena GmbH, Germany) with CoolSNAP and the images were processed using Image-Pro Plus 4.1 software (Media Cybernetics, Silver Spring, MD, USA). Chromosomes were classified as metacentric (m), submetacentric (sm), subtelocentric (st), and acrocentric (a) according to their arm length ratios based on [35].

## Results

### Standard karyotype and Ag-NORs analysis

**Fig 2:**
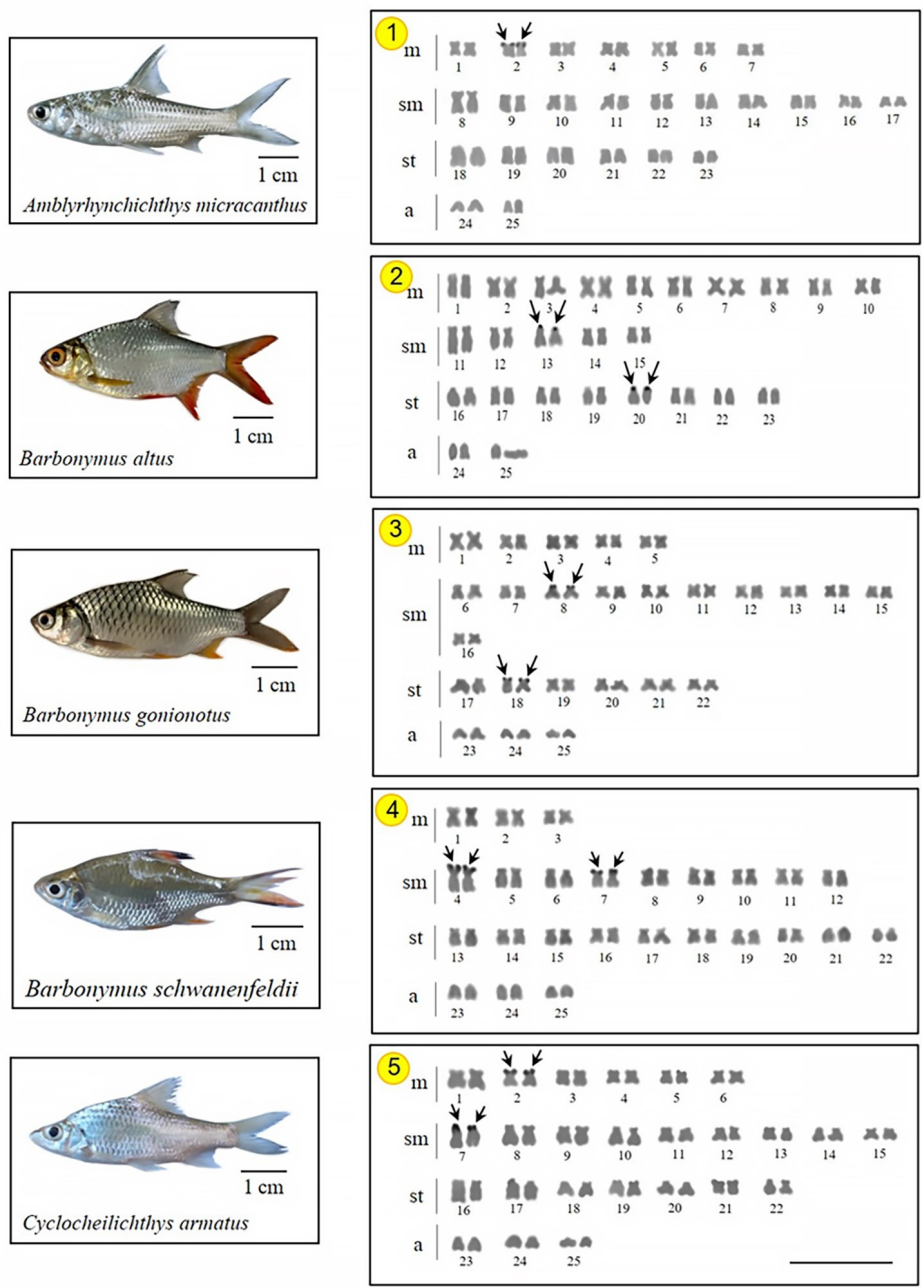
Karyotypes of five Systomini representatives. *Amblyrhynchichthys micracanthus* (1); *Barbonymus altus* (2); *Barbonymus gonionotus* (3); *Barbonymus schwanenfeldii* (4); and *Cyclocheilichthys armatus* (5) karyotypes arranged from Ag-NOR stained chromosomes. The Arrows indicate NOR-bearing chromosomes. Bar = 5 μm.

**Fig 3:**
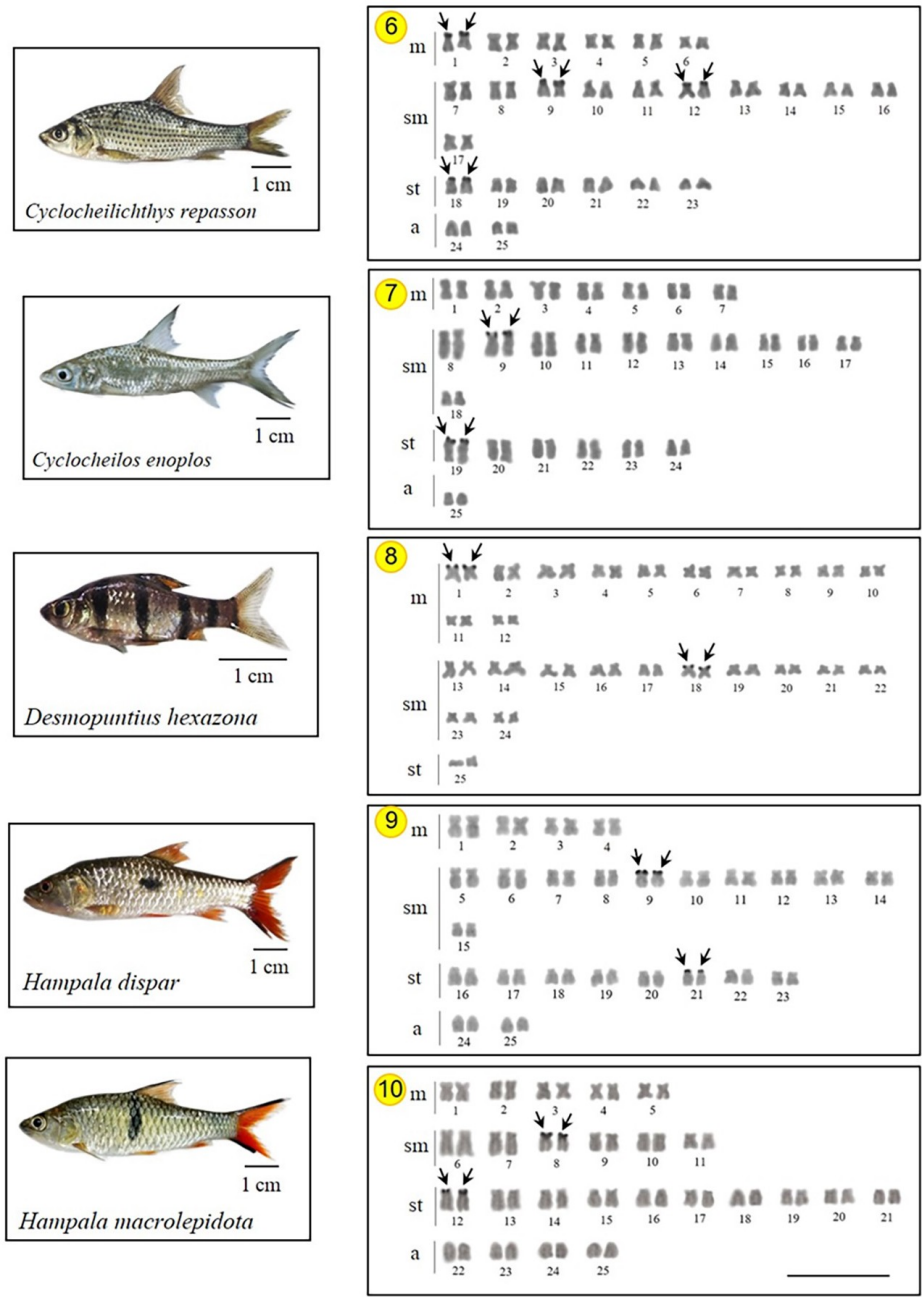
Karyotypes of five Systomini representatives. *Cyclocheilichthys repasson* (6); *Cyclocheilos enoplos* (7)*; Desmopuntius hexazona* (8); *Hampala dispar* (9); and *Hampala macrolepidota* (10) chromosomes arranged in karyotypes from Ag-NOR stain technique. Arrows indicate NOR-bearing chromosomes. Bar = 5 μm.

**Fig 4:**
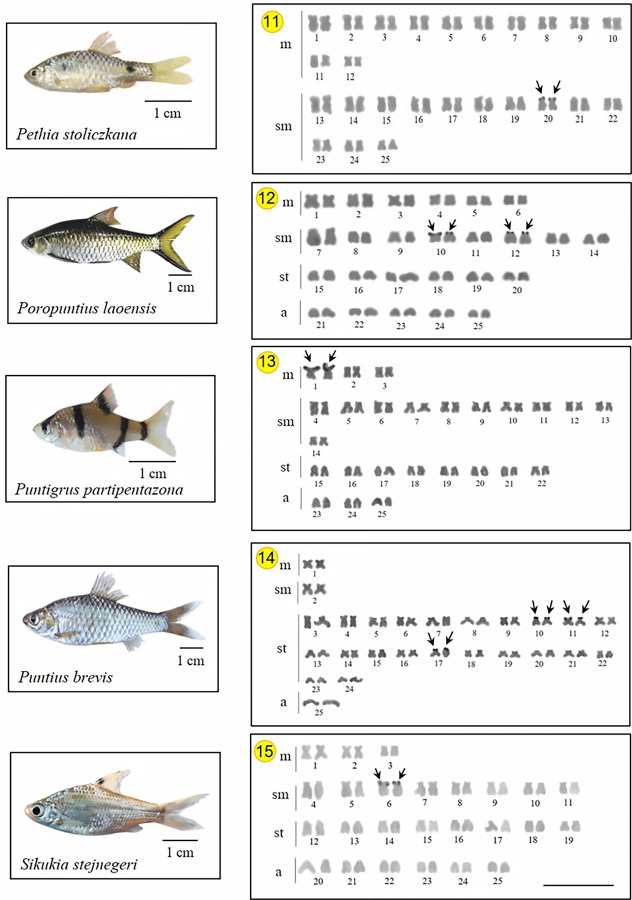
Karyotypes of five Systomini representatives. Chromosomes of *Pethia stoliczkana* (11); *Poropuntius laoensis* (12); *Puntigrus partipentazona* (13); *Puntius brevis* (14) and *Sikukia stejnegeri* (15) arranged in karyotypes from Ag-NOR stain. Arrows indicate NOR-bearing chromosomes. Bar = 5 μm.

As expected, all 15 investigated species presented a diploid number 2n *=* 50, with karyotypes composed of metacentric (m) submetacentric (sm) subtelocentric (st), and acrocentric (a) chromosomes, with the NF ranging 88–100 in both sexes. Two species do not have all chromosome types in their karyotypes, following *D*. *hexazona* without acrocentric ones and *P*. *stoliczkana*, which only harbors meta- or submetacentric chromosomes. Commonly, the Ag-NOR positive sites were observed in the telomeric region of the short arm of 1 (e.g., *A*. *micracanthus*) to 4 chromosome pairs (e.g., *C*. *repasson*). In all species, no numerical or structural polymorphism between the sexes was observed, thus there was no evidence of differentiated sex chromosomes (Figs 2–4).

### Fluorescence in situ hybridization (FISH)-mapping

**Fig 5:**
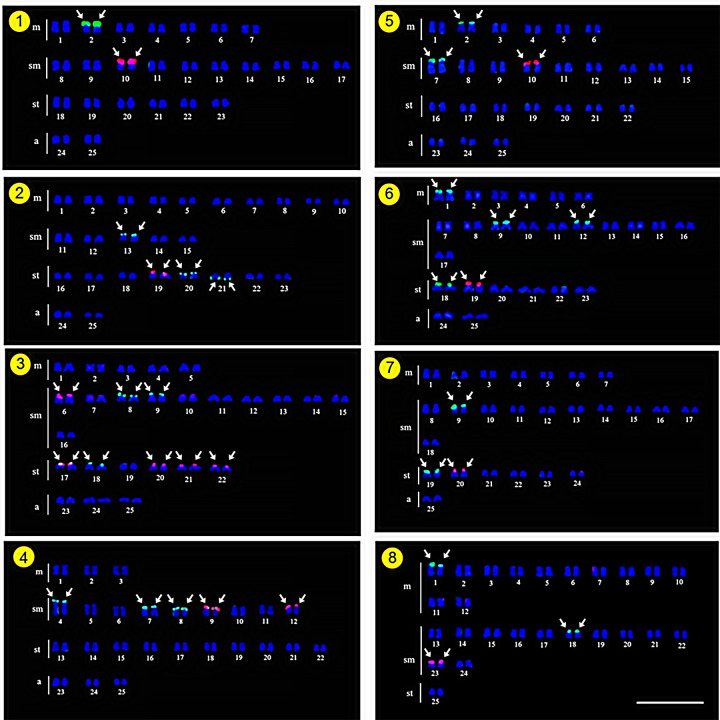
Karyotypes of Systomini species after double-FISH mapping of rDNA sequences. Chromosomes of *Amblyrhynchichthys micracanthus* (1); *Barbonymus altus* (2); *B*. *gonionotus* (3); *B*. *schwanenfeldii* (4); *Cyclocheilichthys armatus* (5); *C*. *repasson* (6) *Cyclocheilos enoplos* (7) and *Desmopuntius hexazona* (8) arranged after double FISH with 5S (red) and 18S (green) rDNAs. Scale Bar = 5 μm.

**Fig 6:**
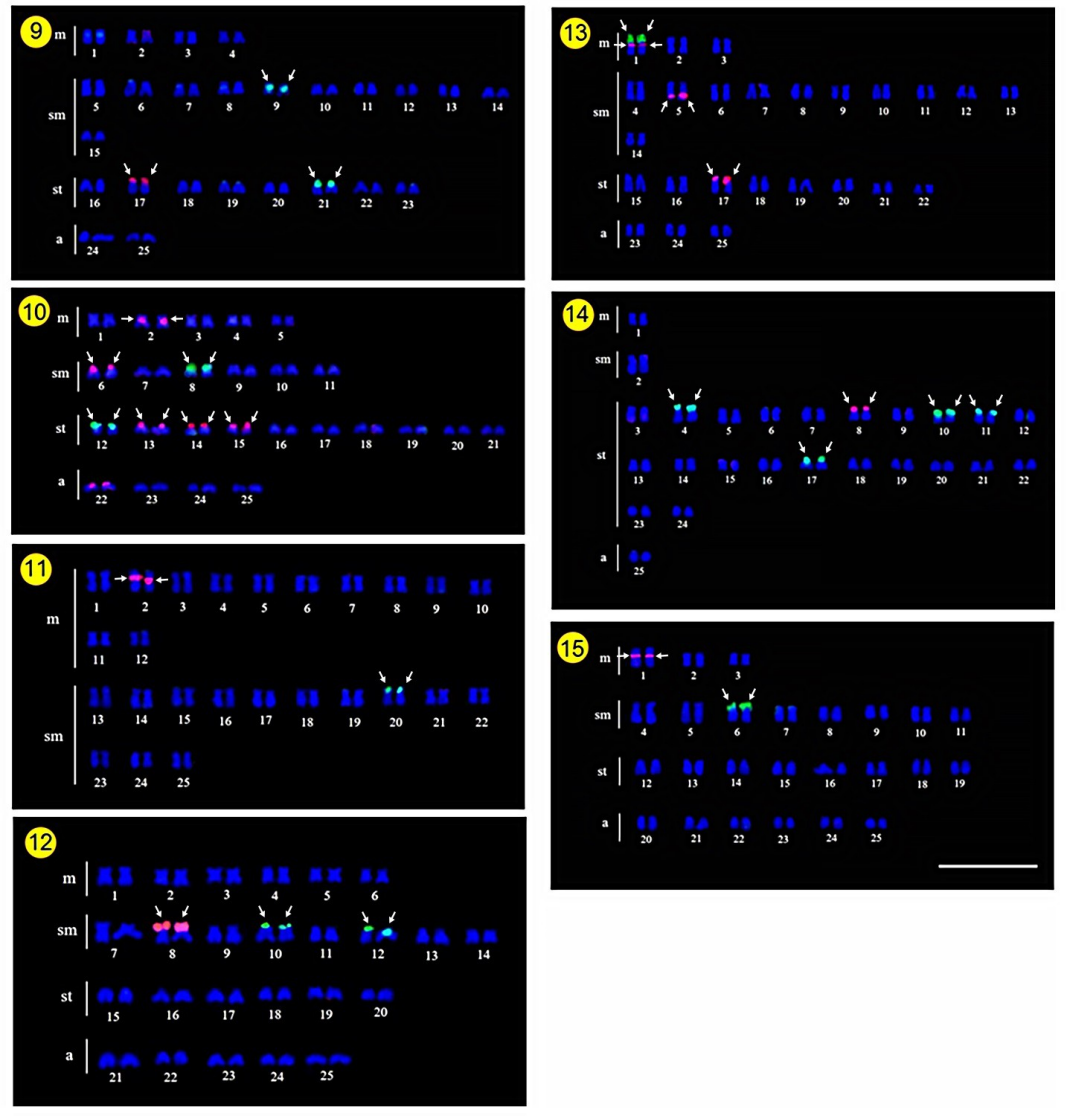
Karyotypes of Systomini species after double-FISH mapping of rDNA sequences. *Hampala dispar* (9); *H*. *macrolepidota* (10); *Pethia stoliczkana* (11); *Poropuntius laoensis* (12); *Puntigrus partipentazona* (13); *Puntius brevis* (14) and *Sikukia stejnegeri* (15) chromosomes arranged after double FISH with 5S (red) and 18S (green) rDNAs as probes. Scale Bar = 5 μm.

**Fig 7:**
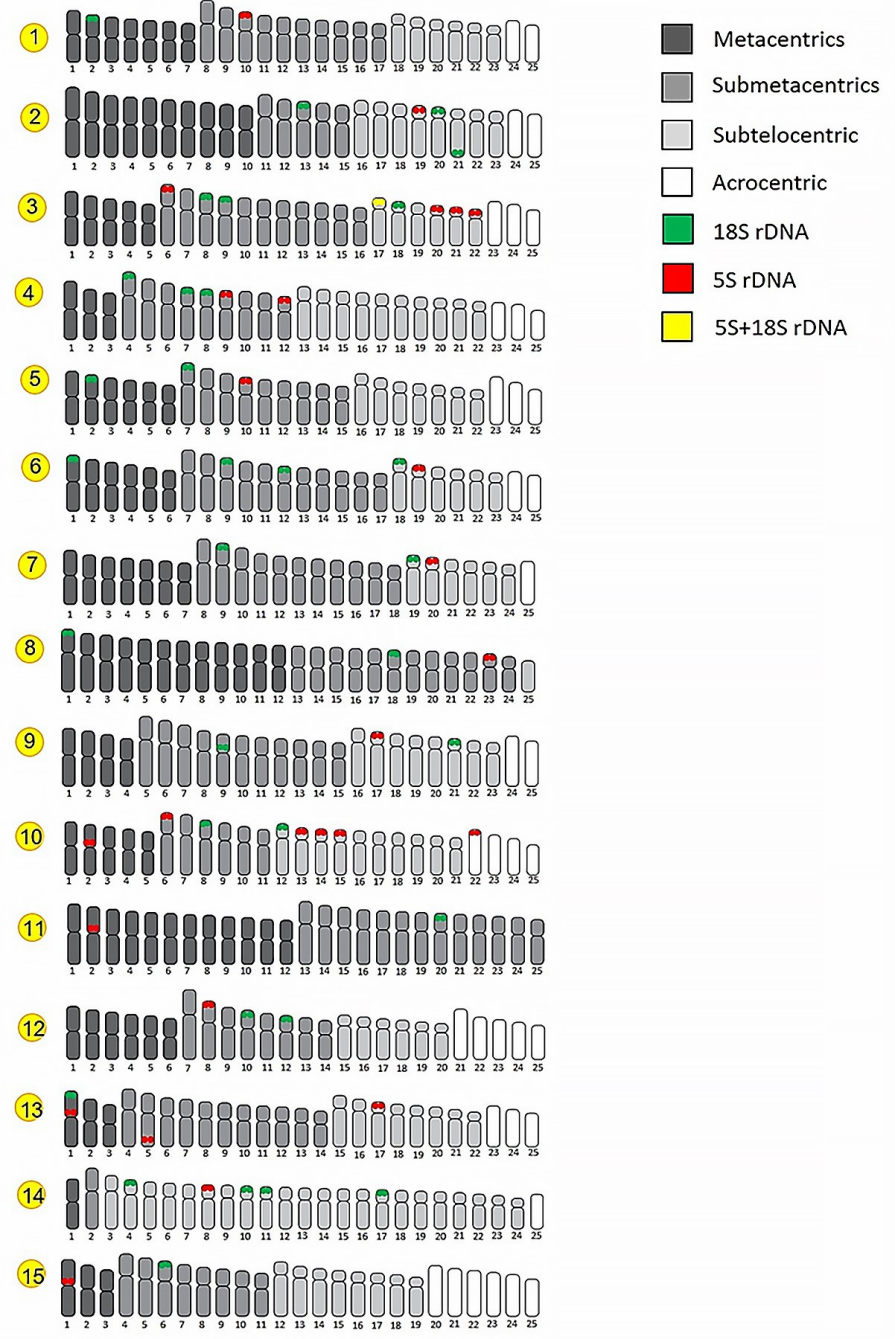
Representative idiograms of the distribution of rDNA sequences in Systomini. Each line corresponds to the representation of the haploid set (n) of a species, following: *Amblyrhynchichthys micracanthus* (1); *Barbonymus altus* (2); *B*. *gonionotus* (3); *B*. *schwanenfeldii* (4)*; Cyclocheilichthys armatus* (5); *C*. *repasson* (6); *Cyclocheilos enoplos* (7); *Desmopuntius hexazona* (8); *Hampala dispar* (9); *H*. *macrolepidota* (10); *Pethia stoliczkana* (11); *Poropuntius laoensis* (12); *Puntigrus partipentazona* (13); *Puntius brevis* (14); and *Sikukia stejnegeri* (15). Red and green circles indicate the position of 5S and 18S rDNA, respectively.

The existence of major ribosomal sites in these Ag-NOR regions was confirmed by FISH with the 18S rDNA probe. The FISH signals coincided with the Ag-NOR regions (Figs 5–7 and Table 2) in the majority of the species studied, with exception of *Barbonymus altus*, *B*. *gonionotus*, *B*. *schwanenfeldii*, and *Puntius brevis* which had additional rDNA loci not identified by the classical Ag-NOR. The 18S probe hybridized to only one chromosomal pair in four species: *A*. *micracanthus*, *P*. *stoliczkana*, *P*. *partipentazona* and *S*. *stejnegeri*, on the short arms of the metacentric chromosome pair 2, submetacentric pair 20, metacentric pair 1, and submetacentric pair 6, respectively. Six species demonstrated positive FISH signals on two chromosome pairs, namely *C*. *enoplos*, *C*. *armatus*, *D*. *hexazona*, *H*. *dispar*, *H*. *macrolepidota* and *P*. *laoensis*. Three positive signals were detected in *B*. *altus* and *B*. *schwanenfeldii*. Furthermore, up to four pairs were detected in *B*. *gonionotus*, *C*. *repasson*, and *P*. *brevis*. 18S rDNA sites were found in telomeric regions of short arms except for the chromosome pair 21 of *B*. *altus* whose signal is accumulated in the telomeric region of its long arms. Moreover, a sub-telomeric distribution of 18S rDNA could be observed at chromosome pair 9 of *H*. *dispar*.

**Table 2:** Cytogenetic and FISH studies on fifteen cyprinids in Thailand (m = metacentric, sm = submetacentric, st = subtelocentric, a = acrocentric chromosomes, NOR = nucleolar organizer region).

Species	Ag-NOR pair (type)	rDNA pair (site)	
		5S rDNA	18 rDNA
*Amblyrhynchichthys micracanthus*	2 (m)	10 (telomeric)	2 (telomeric)
*Barbonymus altus*	13 (sm), 20 (st)	19 (telomeric)	13, 20, 21 (telomeric)
*B*. *gonionotus*	8 (sm), 18 (st)	6,17, 20, 21, 22 (telomeric)	8, 9, 17, 18 (telomeric)
*B*. *schwanenfeldii*	4 (sm), 7 (sm)	9, 12 (telomeric)	4, 7, 8 (telomeric)
*Cyclocheilichthys armatus*	2 (m), 7 (sm)	10 (telomeric)	2,7 (telomeric)
*C*. *repasson*	1 (m), 9,12 (sm), 18 (st)	19 (telomeric)	1, 9, 12, 18 (telomeric)
*Cyclocheilos enoplos*	9 (sm), 19 (st)	20 (telomeric)	9,19 (telomeric)
*Desmopuntius hexazona*	1 (m), 18 (sm)	23 (telomeric)	1, 18 (telomeric)
*Hampala dispar*	9 (sm), 21 (st)	17 (telomeric)	9 (sub-centromeric), 21 (telomeric)
*H*. *macrolepidota*	8 (sm), 12 (a)	2 (sub-centromeric), 6, 13, 14, 15, 22 (telomeric)	8, 12 (telomeric)
*Pethia stoliczkana*	20 (sm)	2 (sub-centromeric)	20 (telomeric)
*Poropuntius laoensis*	10, 12 (sm)	8 (telomeric)	10, 12 (telomeric)
*Puntigrus partipentazona*	1 (m)	1 (sub-centromeric), 5 (interstitial), 17 (telomeric)	1 (telomeric)
*Puntius brevis*	10, 11, 17 (a)	8 (telomeric)	4, 10, 11, 17 (telomeric)
*Sikukia stejnegeri*	6 (sm)	1 (sub-centromeric)	6 (telomeric)

The hybridization with the 5S rDNA probe revealed signals in one chromosomal pair in most species: *A*. *micracanthus*, *B*. *altus*, *C*. *enoplos*, *C*. *armatus*, *C*. *repasson*, *D*. *hexazona*, *H*. *dispar*, *P*. *stoliczkana*, *P*. *laoensis*, *P*. *brevis*, and *S*. *stejnegeri*. In addition, four other species had remarkably increased the number of chromosomes displaying 5S rDNA sequences, namely *B*. *schwanenfeldii* with two, *P*. *partipentazona* with three, *B*. *gonionotus* with five, and *H*. *macrolepidota* with six chromosome pairs. Almost all fish species have shown that hybridization signals of 5S rDNA are abundantly distributed in telomeric regions of the short arm. Pericentromeric regions of chromosome pair 2 of *H*. *macrolepidota*, pair 2 of *P*. *stoliczkana*, pair 1 of *P*. *partipentazona* and pair 1 of *S*. *stejnegeri* were also detected by FISH (Figs 5–7 and Table 2). Moreover, the long arms of the chromosome pair 5 in *P*. *partipentazona* have interstitial signals of the 5S rDNA. In addition, the syntenic arrangement of 5S and 18S rDNAs could be observed in *B*. *gonionotus*. We compiled the distribution of both rDNA probes in cyprinids karyotypes on a comparative idiogram (Fig 7).

## Discussion

### Analysis of karyotypes and Ag-NORs

The use of the 18S rDNA probe confirmed the previous Ag-NOR sites identified of one up to four other sites located in the telomeric regions of distinct chromosomes, characterizing an interspecific variation. Our results showed that the karyotype patterns of 15 fishes in the Systomini tribe are similar to those considered basal and preserved in most Cyprininae. These symplesiomorphies are characterized by a diploid number (2n) equal to 50, as also observed for this subfamily in previous reports [23, 36–40], and most Cyprinidae groups [26, 41–43]. Karyotypes of cyprinids are usually composed of all four chromosome classes, mainly of 2–24 metacentric, 2–26 submetacentric, 2–44 subtelocentric, and 2–8 acrocentric chromosomes, without distinguishable sex chromosomes [23, 37, 38, 44–49]. Sex chromosomes may be present but at an early stage of differentiation that cannot be detected by classical cytogenetic analyses [50], or either by FISH mapping with ribosomal sequences as herein shown. Indeed, we couldn’t observe cytogenetical differences among sexes.

Homologous Ag-NOR variations are found in the species studied here and these variations are common in fish chromosomes, either representing structural polymorphisms [12] or due to genetic regulation of their ribosomal cistrons [15]. The presence of the heterochromatin in these chromosome regions can promote structural alterations as well as may be an important element in the probable genetic control of these cistrons [51]. Our findings demonstrated that NORs can be detected between 1–4 chromosome pairs (Figs 2–4) in most Systomini fish. The single NOR-bearing chromosome pair in *P*. *partipentazona* is consistent with previous investigations [41], while the Ag-NOR pattern previously reported for the genus *Barbonymus*, including *B*. *schwanenfeldii* [52], indicates the NOR exclusive to one pair, which was not equal to the current reported two pairs (Fig 2). Some species had two pairs, including *C*. *enoplos* [45], and *C*. *armatus* consistent with the results of Chaiyasan (2018) [23]. Our mapping also revealed three marked pairs in *Puntius brevis*, which is inconsistent with previous studies [53] that reports one pair of NOR at the telomeric region on the short arm of a subtelocentric chromosome. Differences in the number of Ag-NOR and 18S rDNA sites is a common feature of fish karyotypes, as well as in other vertebrates [54, 55]. The nucleolus organizers regions (NORs) represent the location of genes (loci) responsible for ribosome synthesis (18S, 5.8S, and 28S ribosomal RNA). NORs produce a large number of gene expressions and contain more non-histone proteins than any other chromosome region [56]. It is recognized that the appropriate substance that has an affinity for silver and is stained by this element is a collection of nucleolar argentophilic proteins [57–59]. Nevertheless, certain species could have additional argentophilic proteins outside the nucleolar region, which might additionally stain with silver nitrate and impede accurate Ag-NOR identification. Ag-NOR sites have been thoroughly examined in many species of various groups, including fish [60–62], birds [63, 64], frogs [65], and mammals [66, 67], for example. Besides their simple description as a character of the species, the Ag-NORs have been largely utilized in several other investigations, such as comparative and evolutionary studies, identification of sex chromosome systems [68], and phylogenetic relationships [69]. The development of molecular cytogenetic techniques, especially FISH, made significant progress in chromosomal research possible. Here, the quantity and distribution of ribosomal sequences inside chromosomes are demonstrated using appropriate probes designed for this purpose. This approach, however, does not lessen the validity of Ag-NORs identification because it is still a quick and helpful marker to examine the primary rDNA cistrons and to confirm those that were transcriptionally active during the previous cell cycle interphase [70].

### Chromosomal mapping of 18S and 5S rDNAs

The position of both 5S and 18S rDNA sequences on chromosomes was compiled in the idiograms (Fig 7) and Table 2. Although a high variability in the number of chromosomes carrying the 18S rDNA occurred in *B*. *gonionotus*, *C*. *repasson* and *P*. *brevis*, this sequence is highly conserved regarding its position on the other species. On the other hand, the inverse pattern is found in 5S rDNA sites, in which the loci are located in different pairs in representatives of the Systomini. Two species, *P*. *partipentazona* and *B*. *gonionotus*, have sequences of both 5S and 18S that appear on the same chromosome. In particular, there is a strong hybridization pattern in *P*. *partipentazona* for 18S rDNA probe. this same species, both 5S and 18S rDNAs appeared to be quite conservative and located in the same pair of chromosomes. Moreover, in *B*. *gonionotus*, some but not all 5S loci are coincident with the 18S rDNA loci. Although Ag-NORs sites are productive cytotaxonomic sites in conservative karyotypes, in some cases rDNA may seem limited to identifying specific differences in its location and frequency, especially among species from families with outstanding chromosomal conservation and hence low evolutionary dynamics [71, 72]. The two most frequently repetitive sequences used in fish chromosomal evolution investigations are the 18S and 5S ribosomal genes [73]. The 5S ribosomal DNA (rDNA) is made up of one transcription unit of around 120 base pairs, and non-transcribed spacer regions (NTS) divide each transcription unit from the next [74]. The 18S rDNA probe hybridized to only one chromosomal pair in several species, namely *A*. *micracanthus*, *B*. *altus*, *C*. *enoplos*, *C*. *armatus*, *C*. *repasson*, *D*. *hexazona*, *H*. *dispar*, *P*. *stoliczkana*, *P*. *laoensis*, *P*. *brevis* and *S*. *stejnegeri*. This site is located in the telomeric region of the short arms of that chromosome pair in all species, as also observed in other cyprinids [75]. On other hand, the 5S rDNA probe hybridized in 5, 2, 6, and 3 chromosomes of *B*. *gonionotus*, *B*. *schwanenfeldii*, *H*. *macrolepidota* and *P*. *partipentazona*, respectively. FISH physical mapping in rare situations has revealed a large distribution of 5S rDNA in most of the chromosomes of some species, especially in families with conservative evolutionary patterns [76].

Ribosomal DNAs (rDNAs) represent an important source of information on genome structure and evolution in several vertebrates [77]. In fish, studies with rDNAs demonstrate the chromosome homologies by identifying syntenic groups conserved or rearranged during karyotype evolution [33, 78, 79]. For example, the results of Rossi (2012) [79] studies suggest that the observed high variability of 5S rDNA loci is an effective tool for investigating karyotype differences in Leuciscinae (Cyprinidae) species with conservative 2n. For the genus *Osteochilus* (Cyprinidae, Labeoninae), although also presenting the conserved pattern for cyprinids of 2n = 50, the investigation of rDNAs and microsatellite motifs demonstrate that extensive chromosomal rearrangements occurred along their evolutionary process [75].

The structure of the karyotype and the mapping of the ribosomal DNA sequence demonstrate the evolutionary dynamics of Systomini karyotypes. We demonstrate that 2n = 50 is the common diploid number, following the pattern of Cyprinidae, with variation in the number and position of rDNA sequences. Two species presented a syntenic association of both 5S and 18S rDNAs, an uncommon condition of fish karyotypes. The use of repetitive DNA in combination with other chromosomal analysis procedures is useful for knowledge of the heterochromatic composition of genome and karyotype evolution for a wide variety of fish species, and the results herein described and discussed increased the knowledge on the evolution of the largest freshwater fish family.
